# Flavonoids and Other Phenolic Compounds from Medicinal Plants for Pharmaceutical and Medical Aspects: An Overview

**DOI:** 10.3390/medicines5030093

**Published:** 2018-08-25

**Authors:** Duangjai Tungmunnithum, Areeya Thongboonyou, Apinan Pholboon, Aujana Yangsabai

**Affiliations:** 1 Department of Pharmaceutical Botany, Faculty of Pharmacy, Mahidol University, Bangkok 10400, Thailand; areeya.tho@hotmail.com (A.T.); smart.lady.angel@gmail.com (A.P.); Por.aujana@gmail.com (A.Y.); 2 Department of Botany, Tsukuba Botanical Garden, National Museum of Nature and Science, 4-1-1 Amakubo, Tsukuba 305-0005, Japan

**Keywords:** flavonoid, medicinal and pharmaceutical applications, medicinal plants, phenolics

## Abstract

Phenolic compounds as well as flavonoids are well-known as antioxidant and many other important bioactive agents that have long been interested due to their benefits for human health, curing and preventing many diseases. This review attempts to demonstrate an overview of flavonoids and other phenolic compounds as the interesting alternative sources for pharmaceutical and medicinal applications. The examples of these phytochemicals from several medicinal plants are also illustrated, and their potential applications in pharmaceutical and medical aspects, especially for health promoting e.g., antioxidant effects, antibacterial effect, anti-cancer effect, cardioprotective effects, immune system promoting and anti-inflammatory effects, skin protective effect from UV radiation and so forth are highlighted.

## 1. Introduction

Flavonoids and the other phenolic compounds are commonly known as plant secondary metabolites that hold an aromatic ring bearing at least one hydroxyl groups. More than 8000 phenolic compounds as naturally occurring substances from plants have been reported [1,2]. It is very interesting to note that half of these phenolic compounds are flavonoids presenting as aglycone, glycosides and methylated derivatives [1,2]. These phytochemical substances are presented in nutrients and herbal medicines, both flavonoids and many other phenolic components have been reported on their effective antioxidants, anticancer, antibacteria, cardioprotective agents, anti-inflammation, immune system promoting, skin protection from UV radiation, and interesting candidate for pharmaceutical and medical application [1,3,4,5,6]. Since a few decades ago, the research studies focusing on flavonoids and the other phenolic compounds from medicinal plant species have increased considerably, because of their versatile benefits for human health [1,2,7,8,9,10,11]. Most of the recent reviews focused on one precise aspect of flavonoids or phenolics action on human health.

This work aims to provide an overview of flavonoids and other phenolic phytochems as the potential sources of pharmaceutical and medical applications from the recent published studies as well as some interesting directions for future researches. The key word searches for flavonoids, phenolics, medicinal plant were performed on June, 2018 using Scopus, Google scholar and PubMed. The 351 resulted publications were found and carefully read, in order to find the more recent and non-redundant publications meeting the objective of this work with a few older publications to highlight some necessary points were also used. The 105 selected publications were employed in this review.

## 2. Effects of Plant Flavonoids and Other Phenolics on Human Health Promoting, Diseases Curing and Preventing

### 2.1. Antioxidant Effects

During the production of adenosine triphosphate (ATP) to generate energy for the cells by using oxygen, reactive oxygen species (ROS) and reactive nitrogen species (RNS) are produced as the by-products from these cellular redox reaction. At the balance level, ROS and RNS are beneficial compounds for cellular functions and immune responses, but the unbalance concentration of ROS and RNS will lead to oxidative stress which can cause chronic and degenerative disorders [12,13]. The naturally occurring antioxidant molecules have significantly increased in both usages and research studies; many natural antioxidant compounds have been employed in medical and pharmaceutical products as the substitute compounds for artificial antioxidant ones which have suspected to be one of the major causes for carcinogenesis [14]. Medicinal plants have long been reported as a prospective hub of natural antioxidant compounds, particularly plant secondary metabolites i.e., phenolic compounds and flavonoids which are generated by plant to defend itself or to promote the growth under unfavorable conditions. In addition, functional group arrangement, configuration, substitution, the number of hydroxyl groups were also influenced by antioxidant activity of flavonoids, for example radical scavenging activity and/or metal ion chelation ability [15]. Phenolics and flavonoids are commonly known as the largest phytochemical molecules with antioxidant properties from plants [5,9,10,16,17,18,19].

Oki and his team examined antioxidant activity of anthocyanins and other phenolic compounds from various cultivars of purple-fleshed sweet potato (*Ipomoea batatas* (L.) Lam.), an edible and economic medicinal species in Japan by diphenyl-2-picrylhydrazyl (DPPH) radical-scavenging activity; the obtained results showed the positive correlation between phenolic content and the activity of free-radical scavenging. In addition, chlorogenic acid was the phenolic compounds that acted as dominant DPPH radical-scavenger in “Miyanou-36” and “Bise” cultivars of *I. batatas*, whereas anthocyanins were the dominant DPPH radical-scavengers of “Ayamurasaki” and “Kyushu-132” cultivars [11]. *Bauhinia variegata* L., a medicinal plant that was used in traditional medicine in Pakistan, India and other Asian countries, was studied by Mishra and his group. The researchers found that leaf extracts of *B. variegate* contained flavonoid compounds, and presented antioxidant properties against oxidative damage by radical neutralization, iron binding and reducing power abilities [17]. The antioxidant activity and phytochemical characterization of young and adult cladodes, peel of the fruit and pulp of the fruits from six Spanish Mediterranean cultivars of *Opuntia ficus-indica* (L.) Mill. were analyzed by Andreu and his team. This research team discovered that the significant levels of total phenolic compounds in the best antioxidant cultivar played a significant role against oxidative stress [5]. The antioxidant property and bioactive compounds from the fruits of *Aesculus indica* (Wall. ex Cambess.) Hook, a medicinal plant from temperate regions of Asia i.e., Pakistan, Nepal, India and Afghanistan, were analyzed by the research group of Zahoor; their results indicated that 2-hydroxy-2-phenyle acetic acid (mandelic acid) and 2-(3,4-dihydroxy phenyl)-3,5,7-trihydroxy-4H-Chromen-4-one (quercetin) were the major bioactive molecules with significant antioxidant property to decrease oxidative stress caused by ROS [19]. Furthermore, the rhizomes extracts of *Polygonatum verticillatum* (L.) All., an Indian medicinal plant, were also exhibited antioxidant activity which is associated with the level of phenolic composition [20]. The research group of Meng evaluated the biological activity and phytochemical profiling from the leaves extract of *Camellia fangchengensis* S. Ye Liang and Y.C. Zhong, a wild tea species which local people have been used for green tea or black tea production, that is an endemic tea species in Guangxi province, Republic of China. The acquired results proved that flavan-3-ol oligomers and monomers were the potent antioxidant compounds and abundantly found in this species [6].

Besides the angiosperms or flowering plants, the antioxidant property of phenolic compounds was also reported in gymnosperms, the necked-seed plants. Ustun and his research group studied twig and needle extracts and essential oils of the 5 Turkish *Pinus* species such as *P. brutia* Tenore (Turkish pine), *P. pinea* L. (stone pine or umbrella pine), *P. halepensis* Miller (Aleppo pine), *P. sylvestris* L. (Scots pine) and *P. nigra* J.F. Arnold (European black pine), as well as pycnogenol which is the bark extract from *P. pinaster,* in order to investigate their phytochemical compounds and antioxidant activities by using DPPH and *N*,*N*-dimethyl-*p*-phenylendiamine (DMPD) radical scavenging, ferric-reducing antioxidant power (FRAP), and metal-chelating assays. Their results indicated that pycnogenol had the richest total phenol content, and revealed effective antioxidant effects [21]. Likewise, Apetrei and his collaborators conducted their study on phytochemical compounds and biological activity of *Pinus cembra* L., a native species of Central European Alps and the Carpathian mountains; they discovered that hydromethanolic extract from bark provided higher concentration of total phenolics and flavonoids than that of needle extract. Additionally, the bark extract showed better ability as free radical scavenger [22].

### 2.2. Antibacterial Effect

Interestingly, there are a large number of flavonoids and phenolics which exhibit antibacterial effect; such those compounds can be widely found in non-flowering medicinal plants to the flowering ones. The fern, *Aspleniumnidus nidus* L., contained gliricidin 7-*O*-hexoside and quercetin-7-O-rutinoside that can fight against the 3 pathogens e.g., *Proteus mirabilis* Hauser, *Proteus vulgaris* Hauser and *Pseudomonas aeruginosa* (Schroeter) Migula [23]. Moreover, flavonoid and phenolic compounds are synthesized by various plant groups including many medicinal plant species that are employed in traditional medicine or dietary consumption. An obvious example is nutmeg or *Myristica fragrans* Houtt.; this plant is mostly used traditionally as flavoring agent in Indonesia and other countries in South East Asia [24,25]. However, ethanolic extract of the nutmeg seed which contained 3′,4′,7-trihydroxyflavone showed effective potential against MDR gram-negative bacteria e.g., *Providencia stuartii* Ewing and *Escherichia coli* (Migula) Castellani and Chalmers. [25]. Similarly, *Pseudarthria hookeri* Wight and Arn which has been used as traditional herbal medicine in Africa [26,27] for the treatment of pneumonia, abdominal pains, cough and diarrhea. According to the antibacterial study of this medicinal species, Dzoyem and his team found that flavonoids from this plant showed the highest antibacterial effect against both gram-positive and gram-negative bacteria e.g., *E. coli*, *Klebsiella pneumonia* (Schroeter) Trevisan, *Pseudomonas aeruginosa* (Schroeter) Migula, *Enterococcus faecalis* (Andrew and Horder) Schleifer and Kilpper-Blazand, and *Staphylococcus aureus* Rosenbach; the highest antibacterial activities found in pseudarflavone A and 6-prenylpinocembrin [27]. In addition, Rajarathinam and his group found that *Pseudomonas aeruginosa* (Schroeter) Migula, an important resistant strain that caused many problems in medical treatment can be eliminated by 2-(3′,4′ dihydroxy-phenyl) 3,5,7-trihydroxy-chromen-4-one from the aerial part extract of *Trianthema decandra* L.; this phytochemical compound showed antibacterial activity against this pathogen comparing to chloremphenical, an antibiotic [28]. Flavonoids and other phenolics have also been reported as antibacterial agent against *P. acnes* which are the major cause of skin acne problems. Kaempferol that isolated from the *Impatiens balsamina* L. exhibited potential activity to inhibit the growth of *P. acnes*; its combination with clindamycin and quercetin combined with clindamycin were reported as a better synergic effects [29]. Moreover, flavones which were isolated from the root of *Scutellaria baicalensis* Georgi were proved as potential antibacterial agents against *P. acnes*-induced skin inflammation both in vitro and in vivo models [30]. The study of Hsieh and his team focused on strictinin, the main phenolic compound isolated from the leaves of *Camellia sinensis* var. *assamica* (J.W. Mast.) Kitam which is a raw plant material of Pu’er teas. They discovered that strictinin was a good candidate for antibacterial molecule against this bacteria [31]. Phenolics from kernel extract of *Mangifera indica* L. were also showed anti-acne property to inhibit the growth of *P. acnes* [32].

### 2.3. Anti-Cancer Effect

It is no denying that cancer is one of the major causes of death worldwide; the imbalance and high level of free radicals such as ROS and RNS can also become mutagenic or carcinogenic agents which lead to the cancer development. Chemotherapy is globally employed in cancer treatment, however a large number of drawbacks is its limitation. For example, sometimes the undesired side effects occur during chemotherapeutic treatment. Thus, it is interesting to seek for the alternative treatments for cancer that are no side effects and not so expensive cost. Flavopiridol, a flavonoid-derived drugs from *Dysoxylum binectariferum* (Roxb.) Hook.f. ex Bedd. [Currently the correct scientific name is *Dysoxylum gotadhora* (Buch.-Ham.) Mabb. (http://www.theplantlist.org/tpl1.1/record/kew-2607025)] is an example of anticancer drugs originated from phytochemical compound for lymphomas and leukemia treatments [33,34]. In addition, dietary supplements also play an important role in preventing and curing various kinds of cancer. Phenolic compounds especially flavonoids have long been reported as chemopreventive agents in cancer therapy [2,17,35,36].

Likewise, Danciu and colleagues researched on the phenolic compounds and biological activities of ethanolic extracts from rhizome of *Zingiber officinale* Roscoe and *Curcuma longa* L. which are the core representative species of Zingiberaceae family. This research team proposed the extract of *C. longa* rhizome as the promising source of natural active compounds to fight against malignant melanoma due to its potential anticancer property on B164A5 murine melanoma cell line. The authors also suggested that the increase in anticancer activity was correlated with the increase in amount of polyphenol compounds [37]. Moreover, the results from many biomedical research teams indicated that various kinds of flavonoids can promote apoptosis in various cancer cells [17,35,38]. Quercetin, a flavonol member, is reported as an interesting anticancer substance against prostate and breast cancers [1,38]. Gliricidin7-*O*-hexoside and Quercetin 7-*O*-rutinoside which were the flavonoids isolated from the medicine fern (*Asplenium nidus*) was also purposed as the potential chemopreventive against human hepatoma HepG2 and human carcinoma HeLa cells [23]. According to the intense studied of Hashemzaei and his research group on quercetin and apoptosis-inducing ability both in vitro and in vivo levels. For in vitro studies, they tested anticancer activity of quercetin in 9 cancer cell lines: prostate adenocarcinoma LNCaP cells, colon carcinoma CT-26 cells, pheocromocytoma PC12 cells, human prostate PC3 cells, acute lymphoblastic leukemia MOLT-4 T-cells, estrogen receptor-positive breast cancer MCF-7 cells, ovarian cancer CHO cells, human myeloma U266B1 cells and human lymphoid Raji cells; the obtained results proved that quercetin can significantly induce apoptosis of every tested cell lines at *p* < 0.001 comparing with control group [39]. The in vivo experiments conducted in mouse models i.e., mice bearing MCF-7 tumors and mice bearing CT-26 tumors; the quercetin-treated group exhibited a significant decrease in tumor size and volume at *p* < 0.001 compared to the control group. The survival period of the quercetin-tested animals were also prolonged [39]. Besides, the research team of Clifford conducted their research to evaluate anticancer benefits of quercetin on patient-derived pancreatic tissue and 3 established pancreatic cancer cell lines: primary pancreatic cancer cell line ASANPaCa, AsPC1 and PANC1 to go deeper on the cross talk between quercetin a polyphenol phytochemical compound, microRNAs and Notch signaling in the regulation of self-renewing cancer stem cell divisions [40]. Notch is known as an important gene for signaling receptor encoding, which leads to proper development, the decision of cell fate, cell proliferation and survival [41,42]; it is suggested as a good marker of oncogene and symmetric cell division [43]. Clifford team showed that quercetin can induced miR-200b-3p to regulate the mode of self-renewing divisions of the tested pancreatic cancer [40]. The intense reviewed on genistein and its molecular effects on prostate cancer by Adjakly and his group pointed out that a soy isoflavone genistein inhibited the activation of Nuclear factor kappa B (NF-κB) signaling pathway that is occupied the balance of cell survival and apoptosis, this soy isoflavone could also take its action to fight against cell growth, apoptotic and metastasis processes, including epigenetic modifications in prostate cancer [44]. Curcumin is one of natural phenolic compounds exhibiting anticancer effects towards skin cancers, this phenolic can influence the cell cycle by acting as a pro-apoptotic agent [4]. Abusnina and his team investigated the antiproliferative effect of curcumin on melanoma cancer in in vitro level using B16F10 murine melanoma cells. They showed that curcumin acted as non-selective cyclic nucleotide phosphodiesterases (PDE) inhibitor to inhibit melanoma cell proliferation which is related to epigenetic integrator UHRF1; these researchers also suggested that curcumin occurring in diets might be help to prevent this cancer and contribute in the gene expression via epigenetic control [45]. Interestingly, Hisamitsu group investigated prostate cancer therapeutic potential of curcumin on the inhibitory effect of intracrine androgen synthesis using both in vitro and in vivo models. Their in vitro experiments conducted on human prostate cancer cell lines such as LNCaP and 22Rv1 cells; curcumin decreased the expression of genes evolving in steroidogenic acute regulatory proteins, supporting the decline of testosterone synthesis. Curcumin inhibited proliferation of the selected cell lines in this experiment and induced apoptosis of the cancer cells with dose-dependent response. Their in vivo study on transgenic adenocarcinoma of the mouse prostate (TRAMP) model with 1-month oral administration of curcumin displayed that the phytochemical compound regulate the expression of steroidogenic enzyme, including AKR1C2, and suppressed the growth prostate cancer cells by decreasing testosterone levels in prostate tissues of TRAMP mice [46].

### 2.4. Cardioprotective Effects

The cardioprotective effects from various kinds of phenolics and/or flavonoids occurring in medicinal plants have been investigated from many researches since many decades ago [1,47,48,49,50,51,52,53,54,55,56,57]. The comprehensive review of Razavi-Azarkhiavi and his team illustrated cardioprotective role of various phenolic compounds against cardiotoxicity of doxorubicin which is the extensively used anticancer medicine for lymphomas, leukemia and breast cancers in clinical application that contains vulnerable side effect as cardiotoxicity such as pericarditis, arrhythmias, myocarditis, and acute heart failure [52]. They found that antioxidant phenolics have been recommended as a promising approach to reduce adverse effects of this anticancer drug; many phenolic and flavonoid compounds have been studied and reported their cardioprotective properties via various mechanisms including inhibition of ROS generation, mitochondrial dysfunction, apoptosis, NF-kB, p53, and DNA damage both in vitro, in vivo, and clinical studies. Razavi-Azarkhiavi team also found that many flavonoid and phenolics i.e., kaempferol, rutin, luteolin and resveratrol showed their efficacy against doxorubicin-induced cardiotoxicity, but do not affect on the antitumor activity of this medicine [58,59,60]. The most interesting reported compound was isorhamnetin. Because, it provided cardioprotective effect against cardiotoxicity of doxorubicin, and potentiated the anticancer efficacy of this drug [52,61]. Recently, there is the research on phenolic composition from methanolic extracts of the aerial parts of the two medicinal plants in Poland: *Centaurea borysthenica* Gruner and *C. daghestanica* (Lipsky) Wagenitz [At present, the corrected scientific name of this plant is *Centaurea transcaucasica* Sosn. ex Grossh. (http://www.theplantlist.org/tpl1.1/record/gcc-95497? ref=tpl1)] were analyzed together with their protective effects on cardiomyocytes treated with doxorubicin [53]. The obtained results from oxidative stress, cell viability, and mitochondrial membrane potential tests displayed their cardioprotective activity of both *C. borysthenica* and *C. daghestanica* extracts on rat cardiomyocytes treated with doxorubicin anticancer drug. According to this study, they found an attractive point that *C. daghestanica* methanolic extracts did not affect on efficacy of doxorubicin in this experiment [53].

In addition, the research group of Alhaider evaluated the cardioprotective potential of *Phoenix dactylifera* L. or date palm in English name or Nakl in Arabic name. The total flavonoid, total phenolic, in vitro antioxidant capacity and in vivo rodent myocardial infarction models with fruit extracts from 4 different varieties of date palm in eastern provision of Saudi Arabia were confirmed. The high concentrations of phenolics and flavonoids were detected in the fruit extracts that contributed the potential antioxidant activities and high cardioprotective effect against various induced factors in vivo myocardial infarction models by mobilizing the circulating progenitor cells from both bone marrow to the site of myocardial infraction, in order to promote tissue repairing from ischemic injury [56]. Syama and his colleagues evaluated the major phenolic acids and flavonoids from the different fractions of seeds extract from *Syzygium cumini* (L.) Skeels, and their cardioprotective potential in in vitro H9c2 cardiac cell lines such as tertiary butyl hydrogen peroxide induced oxidative stress, LDL oxidation, HMG-CoA reductase and angiotensin converting enzyme modulation. The major phytochemical compounds from the analyzed fractions were gellagic acid, syringic acid, gallic acid, ferulic acid, cinnamic acid and quercetin. These fractions attenuated oxidative stress in H9c2 cardiomyoblasts and molecular docking demonstrated the positive correlation between the major phytochemical compounds and key enzymes for preventing cardiovascular diseases i.e., angiotensin converting enzyme [57]. Moreover, the research group of Garjani investigated the potential of aerial parts extract from *Marrubium vulgare* L., a medicinal plant from Iran focusing on its cardioprotective effects against ischemia-reperfusion injury in vivo Wistar rat model. They determined total phenolic and flavonoids content of aqueous fraction of the extract, and their effect on ischemia-reperfusion injury of the rat hearts using Langendroff method; the obtained result proved that aqueous fraction from *M. vulgare* consisting of cardioprotective potential against this cardiac injury [48]. Aspalathin and phenylpyruvic acid-2-*O*-β-d-glucoside, the two of the major compounds from *Aspalathus linearis* (Burm.f.) R. Dahlgren were demonstrated as potential protective compounds to protect myocardial infarction caused by chronic hyperglycemia [49]. Likewise, puerarin is a potential isoflavones that was reported as an interesting candidate for cardioprotection by protecting myocardium from ischemia and reperfusion damage by means of opening the Ca^2+^-activated K^+^ channel and activating the protein kinase C [51]; this research team conducted their study using in vivo Sprague–Dawley rats model. Tian and his group compared the cardioprotective effects between polyphenolic extracts from apple peel and apple flesh in in vivo mice model with cardiovascular risk factors; they found that the extracts of apple peels exhibited better cardioprotective ability than that of apple flesh in mice model [54]. This may probably due to the higher amount of both total phenolics and total flavonoids consisting in polyphenolic extracts from apple peel.

### 2.5. Immune System Promoting and Anti-Inflammatory Effects

Medicinal and pharmacological agents, nutrients, pollutants and other environmental factors play a necessary role in the human immune system. A large number of flavonoids and other phenolics have been proved their noteworthy effects on immune system function and inflammatory processes [62,63]. Quercetin, apigenin, hesperidin and luteolin were reported as flavonoids containing potential anti-inflammatory effects [1]. The research group of Rupasinghe examined the anti-inflammatory properties of Canadian medicinal plant extracts, *Lonicera caerulea* L. or haskap berry in various cultivars focusing on pro-inflammatory cytokines using in vitro human monocytic cell line THP-1 derived macrophages which stimulated by lipopolysaccharide. Borealis cultivar of Haskap berry presented the highest phenolic, flavonoid and anthocyanin content (*p* < 0.05), and exhibited comparable anti-inflammatory effects to diclofenac which is a COX inhibitory medicine [64]. In addition, the synergistic effects on immune and health promoting properties of bioactive compounds and probiotic bacteria are also currently interested by the scientists. For example, the study of Sisto’s group to investigate effect of *Lactobacillus paracasei* culture filtrates and *Cynara scolymus* L. or artichoke phenolic extract from edible part of its fresh buds on cytokine producing by dendritic cells. The experimental result pointed out the interesting anti-inflammatory effect of a culture filtrate obtained after probiotic *L. paracasei* strain growing in the media supplemented with artichoke phenolic extract [65]. Moreover, the anti-inflammatory activity of polyphenolic compounds in *Gaillardia grandiflora* Hort. ex Van Houte and *Gaillardia pulchella* Foug from Egypt were reported with nontoxicity test in in vivo mice model; the newly reported compound, 8-hydroxyapigenin 6-*O*-β-d-apiofuranosyl-(1′′′→6”)-*C*-*β*-d-^4^C_1_-glucopyranoside, from *G. grandiflora* and other known compound i.e., luteolin 6-*C*-β-d-^4^C_1_-glucopyranoside 8-methyl ether, schaftoside, isoorientin, apigenin 6-*C*-β-d-^4^C_1_- glucopyranoside 8-methyl ether, 6-methoxyluteolin isovitexin and hispidulin were also isolated and tested in this research [66]. The inflammatory inhibition ability both tumor necrosis factor- (TNF-) and interleukin-6 (IL-6) of polyphenol fractions from sixteen cultivars of Chinese blueberries including 14 commercialized ones such as Bluecrop, Bluesource, Berkeley, Brigitta, Duke, Darrow, Misty, Northblue, Northland, Northcountry, O’Neal, Patriot, Reka and Southgood from China were employed in the study of Ma and his team. Their anti-inflammatory effect of these blueberry samples were tested using lipopolysaccharide induced RAW 264.7 macrophages; anti-inflammatory potential of the polyphenol fractions were in the same trend of their phenolic acid contents [67]. Likewise, anti-inflammatory activities of two medicinal plant species: *Bidens engleri* O.E. Schulz from Asteraceae family as well as *Boerhavia erecta* L. from Nyctaginaceae family were tested in various fractions and evaluated their total phenolic and total flavonoid contents [68]. This research team found that dichloromethane was the highest potential solvent to extract flavonoid compounds in both species and this fraction also exhibited anti-inflammatory effect via COX-2 and LOX-15 inhibition. Macrophages play an important role in controlling the switches of immune system by means of maintaining the balance of pro-inflammatory and anti-inflammatory activities. Dugo and his team proved that polyphenol extract from roasted cocoa beans (*Theobroma cacao* L.) significantly lowered pro-inflammatory cytokines secretion in in vitro THP-1 cells, as well as suppressed inflammation by promoting oxidative pathways, which lead to the increase in oxygen consumption by mitochondria and ATP production via oxidative phosphorylation [69]. Additionally, Lopes and his team characterized phenolic composition of *Lavandula pedunculata* (Mill.) Cav. samples from various different geographical origins in Portugal, and compared their bioactive activities in aqueous and hydroethanolic extracts. The obtained results pointed out that the *L. pedunculata* hydroethanolic extract from Alentejo area exhibited highest anti-inflammatory activity in rat RAW 264.7 macrophages by inhibiting nitric oxide production [70].

It is known that COX-2 syntheses prostaglandin E2 is an endogenous pain-producing substance, while COX-1 is a house-keeping enzyme. According to the molecular mechanism of some anti-inflammatory medicines which inhibit both cyclooxygenase-2 (COX-2) and COX-1 enzymes. Consequently, the medicines that inhibits both COX-1 and COX-2 concurrently can cause adverse side effects i.e., renal dysfunction or gastrointestinal bleeding. Therefore, the researchers have challenged to seek for the better candidate for drug development; some phenolics have been reported as the selective inhibiting compounds toward COX-2 expression. An interesting example is the study Ma and his team which aimed to validate the potential and mechanisms of polyphenols from inner bark of *Tabebuia avellanedae* Lorentz ex Griseb [Currently, the corrected species name of this plant is *Handroanthus impetiginosus* (Mart. ex DC.) Mattos; http://www.theplantlist.org/tpl1.1/record/kew-317146], a medicinal plant with extensively use as folk medicine in Central and South America, as an anti-inflammation agent without undesirable side effects from COX-1 inhibition. This work was conducted using in vitro free fatty acid-stimulated macrophage cell lines and combined molecular docking to investigate the interactions between the phenolic compounds and COX-2; the obtained results illustrated anti-inflammatory effects of phenolics from this medicinal plant to regulate macrophages by targeting COX-2 activity inhibition without any action on COX-1 activity [71]. Furthermore, phenolics and flavonoids from bark of *Vitex peduncularis* Wall. ex Schauer, a herbal drug, were characterization together with their anti-inflammatory activity by the research group of Ferreres. They found high content of apigenin, *C*-rhamnosyl flavones and luteolin derivatives in this methanolic bark extract which reduced nitric oxide levels in macrophages and significantly inhibited the activity of phospholipase A2, a mediate enzyme in inflammatory processes [72]. Additionally, Lu and his research group optimized the ethanolic rhizome extract of astilbin, a dihydroflavonol, from *Smilax glabra* Roxb and evaluated its anti-inflammatory effects in in vitro lipopolysaccharide-induced RAW264.7 macrophages. Their results pointed out that astilbin significantly suppressed nitric oxide production, tumor necrosis factor-α (TNF-α), mRNA expression of inducible nitric oxide synthase and TNF-α in the tested cells [73]. Recently, the isolated astilbin flavonoid from rhizome of *S. glabra* in China and its anti-inflammatory potential was also investigated by Dong and his group in in vivo complete Freund’s adjuvant-induced adjuvant arthritis rats (AA rats) model. Their results showed noteworthy inhibitory properties of astilbin on TNF-α, IL-1β as well as IL-6 mRNA expression; serum cytokine levels of TNF-α, IL-1β, and IL-6 were also decreased in treated AA rats. They also proved that oral treatment of astilbin daily at 5.3 mg/kg can reduce joint damage in hind paw of the animal model; this therapeutic properties of astilbin flavonoid on the inhibition of cytokines production and reduction of inflammatory response in in vivo AA rats model were effective as equal as leflunomide, the frequently used antirheumatic drug [74]. Moreover, the double-blind, randomized, placebo-controlled clinical trial on inflammation of ferulic acid, an abundant phenolic compound from various plant including edible medicinal plant and cereal grains, were evaluated in hyperlipidemic subjects by the research team of Bumrungpert. They randomly divided hyperlipidemia subjects into 2 groups i.e., treatment group (*n* = 24) with ferulic acid 1000 mg daily and the control group (*n* = 24) with a placebo for six weeks; ferulic acid supplementation significantly decreased in the inflammatory markers with statistic different comparing with the control group [75].

### 2.6. Skin Protective Effect from UV Radiation

Overexposure to ultraviolet (UV) radiation can harm to skin. It induces extensive production of reactive oxygen species (ROS) and eventually causes skin damages [76]. However, there are several strategies applicable for skin protection. Phytochemical compound, especially phenolics and flavonoids is one of the most interesting choices that exhibits beneficial effects on UV-irradiated skin [77,78,79]. Flavonoids have photoprotective effects that are antioxidant properties by their capacity to chelate iron which can damage lipid and protein on cell membrane, and modulate several signaling pathways, for example, inhibit xanthine oxidase which is considered as a source of ROS that contributes to oxidative stress [80,81]. Several phenolic compounds are reported as potential antioxidant molecules for treatment of various skin disorders including diseases which caused by UV radiation [4,78].

Apigenin is a major flavones with skin protective effect from UV light; this flavone can be found in many edible medicinal plants or plants-derived beverages e.g., red wine, beer and chamomile tea [82,83]. Quercetin is a flavonols which can be found in onion skin, apple peel and *Hypericum perforatum* L. leaves [84]. Topical application with quercitin effectively inhibited UVB-induced skin damage in hairless mice [85]. In addition, *Ginkgo biloba* L. extract (EGb 761) that contains a lot of quercetin derivatives had an ability to decrease sunburn symptoms UVB-induced skin in in vivo study using UVB irradiated-skin mice model; the results indicated that oral intake of EGb 761 may act as a protective and therapeutic agent [86]. Silymarin, a standardized extract of flavonolignans from the milk thistle (*Silybum marianum* (L.) Gaernt.) fruits contains silybin, a major active component [87]. The topical treatment with silymarin stimulated the repair of UVB-induced DNA damage that leads to the prevention of apoptosis in UVB-exposed human epidermal keratinocytes as well as fibroblasts in in vitro study [88]. Genistein is a soybean isoflavone that was also reported as photoprotective molecule against photocarcinogenesis by inhibiting UV-induced DNA damage in human skin equivalent in vitro model [89]. Moreover, Wang and his team examined effect of genistein in human dermal fibroblasts on UVB-induced senescence via the mechanism of oxidative pathway; they found that genistein was able to maintain activities of antioxidant enzymes and modulate mitochondrial oxidative stress [90]. Equol is known as an isoflavonoid metabolite from isoflavone daidzein or genistein producing by gut microflora [91,92]. An in vivo study in hairless mice reported that topical application with equol prior to UV-irradiation can prevent UV-induced erythema-associated edema, immunosuppression and skin cancer by acting as a sunscreen and inhibiting DNA photodamage [92]. Additionally, the study of Choi and his group to evaluate skin protective effects of spent coffee ground on ultraviolet UVB-induced photo aging in in vivo hairless mice model showed that topical application of spent coffee ground extracts consisting of flavonoids and caffeine which were able to protect mouse skin by down-regulating of matrix metalloproteinases [93]. Interestingly, the research team of Kano investigated protective effect of isoflavones from fermented soymilk products on photodamage in the skin of ovariectomized hairless by oral administration for 28 days. The results indicated the increases of isoflavone concentration on mice skin and in their blood can effectively scavenge reactive oxygen species generating by UV irradiation, and also exerting estrogenic activity, resulting in photoprotective effect on skin of the animal model [94].

## 3. Naturally Occurring Plant Phenolics and Flavonoids for Menopausal and Post-Menopausal Women

There is pros and cons between using synthetic chemical compounds and phytochemical substances in pharmacy and medicine. Synthetic substances or medicines are easy and quickly to produce in large scale of drug development process and modify as many forms of consumption for patients. Conversely, many non-natural (or synthetic chemical compounds) cause several undesirable side effects, particularly long-term treatments [95]. Some synthetic medicines were not accept for clinical treatments because of their harmful side effects. An obvious example is synthetic estrogen which was commonly used in menopause women for hormone replacement therapy a few decades ago. This synthetic chemical compounds could work well to reduce menopause symptoms, long before there were a number of researches discovered its unwanted side effects i.e., an increase in the risk of breast, uterus and ovarian cancers [95,96,97].

Estrogen is a sex hormone mainly responsible for reproductive functions and the menstrual cycle of women. In postmenopausal women, estrogen is depleted due to the failure of the response of ovary to pituitary. When the level of estrogen decreases, it leads to many postmenopausal symptoms including cardiovascular disease. In particular, postmenopausal woman who also has metabolic syndrome (MetS) will increase in the risk of cardiovascular disease [98]. The research study of Squadrito and his group showed that flavonoid supplementation can also improve cardiovascular function in postmenopausal woman with metabolic syndrome [99]. Genistein is an obvious example of interesting choice of flavonoid phytoestrogen for improving endothelial functions in postmenopausal women with MetS [100]. Gregorio and his research team investigated the effects of genistein supplement on cardiac function of postmenopausal women with MetS; postmenopausal women patients with type-2 Diabetes mellitus and free from previous cardiovascular disease 120 subjects were employed in this study [98]. The patients were equally divided into 2 groups: Genistein supplementation group and control group who have got placebo by using a computer-generated double-blind randomization. The result indicated that genistein can improve the cardiac function in postmenopausal women with MetS [98].

The decrease of estrogen leads to postmenopausal bone loss or osteoporosis. Morabito research team found that genistein can be used as hormone-replacement therapy (HRT) for preventing osteoporosis in postmenopausal women [101]. This research team aims to compare the effect of genistein phytoestrogen with HRT (estrogen and its derivatives). The study conducted on 90 healthy women between 47–57 years who had bone mineral density at femoral neck of <0.795 g/cm^2^. The 90 participant subjects were randomly and equally divided into three groups: Genistein, HRT and control group treating with placebo continuously for 1 year. The result indicated that using genistein in postmenopausal bone loss is more effective than HRT, because the undesirable side effects were not found in genistein group. Kruger and his team investigated the effects of bone turnover and the change in microflora between the groups of healthy New Zealand post-menopausal women who received daily isoflavone supplementation (daidzein and genistein) alone and those who consumed green kiwifruit combined with isoflavones for 4 months; their results indicated that the second group of post-menopausal women significantly improved bone health [102]. Nevertheless, the minimum and optimum dose are the essential point to concern. The concreted example can be seen in the study of Kaczmarczyk-Sedlak and his research group; their results indicated that moderate dose of isoflavane glabridin from root of *Glycyrrhiza glabra* L. or licorice plant showed no effect on bone loss in ovariectomized rats, an in vivo model of osteoporosis from estrogen deficiency in postmenopausal women [103]. The secondary metabolites discovered in medicinal plants such as flavonoids and other phenolics may avoid the negative side effect of synthetic medicines, because they must accumulate within the cells and tissues of living organisms [71,95,96]. Moreover, many medicinal herbs contain novel or valuable secondary metabolites with different biological properties, and a huge numbers of them are waiting for discovery.

## 4. Profiling Works and the Survey of Flavonoids and Other Phenolics from Medicinal Plants

For over four million years, flavonoids and other phenolic substances from medicinal herbs have been used or consumed by humans so as to live healthy and fight against undesirable diseases [1,22,51]. As a plant secondary metabolites, flavonoids and the other phenolics are found in several plant species, type and amount of the chemical components are vary depending on species and affecting by environmental factors i.e., mineral at the growth locality and geographic origin [70]. Nowadays, there is a significant increase in the number of research on potential of medicinal plant species for pharmaceutical and medical purposes focusing on natural phenolic compounds and flavonoids [1,4,9,16,18,51,63,71,73,104].

Though many flavonoids and other phenolic compounds were examined from medicinal plant species, a large amount of native or endemic medicinal herbs are still waiting for being survey and observing their novel compounds from profiling works. These processes are an indispensable step to promote the progression in drug discovery and development using phytochemical compounds. Asian region is well-known as one of the greatest hotspot of plant biodiversity including medicinal species, especially Japan, China, Thailand and related areas in tropical and sub-tropical regions [6,54,67,74,104,105]. However, a comprehensive profiling of many medicinal plants and functional evaluation of their chemical compound has not been completely conducted. According to our intense review in more than one hundred scientific publications, it is clear that the different parts of medicinal species such as floral parts, leaves, stems, root or rhizome consisting of different types and amount of phenolics and flavonoids. Furthermore, the harvesting season, cultivar and variety of the targeted plant species should be accounted and compared in phytochemical profiling works.

## 5. Future Perspectives and Interesting Directions for Future Researches

(1) The low cost of medicines and many other medical products is very important to allow all people to access to the drug. Consequently, the flavonoid and phenolic compounds which are abundant found in a large number of plant may possible be an interesting choice of molecules for drug and medical product development.

(2) In the same species of medicinal plants, the different cultivars may provide different amount of flavonoid and phenolic compounds as well as the biological activities. Thus, the cultivars of medicinal plant should be taken into account for the future medical and pharmaceutical research studies.

(3) The geographic areas of raw plant material should also be analyzed and compared in the future research. Since the environmental factors e.g., nutrients and mineral in soil are also effect on the quality and quantity of phytochemical compounds in some species of medicinal plant as discussed in this work.

(4) Not only local medicinal plant species but also the wild or endemic species are interesting for the future studies, in order to discover the novel phytochemical compounds to increase the alternative sources of raw material for medical and pharmaceutical applications.

(5) The molecular mechanism and signaling pathway of many known flavonoid and phenolic compounds are need to be done in the future, so as to apply this knowledge to the drug development processes.

(6) The need of purified compounds to confirm data obtained with the plant extracts.

(7) Epidemiological and in vitro studies are sometimes contradictory. In part because of the calculation of the intake based on general table estimated content without taking into account genetic variation among cultivars, geographic variations and so on. Also because of the possible need of metabolization by gut microflora for activation. Those of gut microflora are not taken into account, and quantification of the active circulating forms was not evaluated most of the time.

## 6. Conclusions

To recapitulate, the use of phenolic compounds and flavonoids are the potential candidate of bioactive agents in pharmaceutical and medicinal sectors to promote human health, prevent and cure various diseases. In order to discover and progress these alternative choice of using phytochemical compounds, the survey of medicinal plants together with intense profiling research needs to be done. The targeted compounds should be employed in biomedical and pharmaceutical research ranging from in vitro, in vivo, and clinical trial step to evaluate the safety, efficacy and also the side effects of the tested candidate compounds.
